# Protein quality and allergenicity assessment of chia seeds (*Salvia hispanica*): a molecular perspective on novel food safety

**DOI:** 10.3389/ftox.2026.1735718

**Published:** 2026-04-20

**Authors:** Luisa Calcinai, Sara Cutroneo, Ilaria Puxeddu, Simon Dirr, Özlem Özmutlu Karslioglu, Tullia Tedeschi

**Affiliations:** 1 Department of Food and Drug, University of Parma, Parma, Italy; 2 Immuno-allergology Unit, Clinical and Experimental Medicine, University of Pisa, Pisa, Italy; 3 Institute of Food Technology, University of Applied Sciences Weihenstephan-Triesdorf, Freising, Germany

**Keywords:** allergenicity assessment, chia seeds, HR-MS identification, novel food, protein quality

## Abstract

**Purpose:**

This study aimed to assess the protein quality and allergenic potential of chia seeds (*Salvia hispanica*), introduced in European market as a novel food (Regulation European Union 2015/2283) in 2019. Although chia is increasingly used as a plant-based ingredient, information regarding its protein profile and sequences is still limited. It follows that, without this essential knowledge, information of its allergenic potential is also lacking. Therefore, this work pose itself as a first building block, providing a detailed molecular characterisation of chia proteins and evaluating their allergenic potential and IgE cross-reactivity with other known allergens, such as sesame (*Sesamum indicum*).

**Methods:**

Three chia flour samples—partial defatted flour as reference, standardised partial defatted flour, and protein concentrate—were analysed. Protein content was determined by Kjeldahl method. Protein quality was evaluated based on the amino acid profile and the estimation of amino acid score. Proteins were identified by SDS-PAGE through comparison with existing literature and then confirmed by in solution tryptic digestion coupled with high-resolution mass spectrometry analysis. Allergenicity was assessed by *in silico* sequence homology analysis with characterised sesame linear epitopes and *in vitro* immunoblotting using sera from sesame-allergic patients.

**Results:**

The protein content ranged from 25% to 26% in the raw materials to 56% in the concentrate. Furthermore, SDS-PAGE profiles were comparable between samples, confirming the effectiveness of the extraction method applied. All samples showed balanced amino acid profiles and amino acid scores above one, meeting FAO/WHO requirements for adults and children. The main proteins identified in chia were 11S and 7S globulins, albumins and oleosins. The identified chia peptides were used to obtain a coverage of these with sesame protein sequences, confirming the attended homology. The potential cross-reactivity with sesame proteins, primarily retrieved from the literature, was then confirmed *in vitro*. IgE-binding was detected for major chia proteins, such as 11S and 7S globulin, and 2S albumin endorsing the attended cross-reactivity with sesame proteins.

**Conclusion:**

This study provided insights on the effectiveness of the extraction method applied on chia protein quality, which is essential for their inclusion in balanced food formulations. The approach used for assessing allergenicity also highlighted that the level of molecular and immunological knowledge can differ among novel foods, making it challenging to define a general methodological framework for evaluating their allergenic potential and cross-reactivity risks. These results can be useful both as starting point for the inclusion of protein extracts from chia seeds as safe ingredient, while also highlighting the current lack of comprehensive molecular characterization—including incomplete sequence data and uncharacterized potential epitopes—which limits the full assessment of their allergenic risk.

## 1 Introduction

The search for sustainable and nutritionally balanced sources of protein is gaining attention in the food industry, particularly in the development of plant-based alternatives. Conventional animal proteins are increasingly challenged due to their significant environmental footprint, as well as ethical and health concerns. In this context, plant-based and other alternative proteins are emerging as strategic components of global food security and climate-resilient food systems (Malila et al., 2024). Chia seeds (*Salvia hispanica*), a member of Lamiaceae family, are an ancient pseudo-cereal and new functional food with potential health benefits. They have also proven to be a novel oilseed crop for the Mediterranean region (Muñoz et al., 2013; Sosa and Salvia hispanica, 2016). Chia seeds are known for their high protein content (approximately 18%–24%). Amino acid profiling has revealed the presence of all ten essential amino acids, with particularly high levels of arginine, leucine, phenylalanine, valine, and lysine. In addition, chia proteins contain substantial amounts of non-essential amino acids, especially glutamic and aspartic acids, along with alanine, serine, and glycine, which contribute to cellular health and metabolic functions (Kulczyński et al., 2019). Chia seeds are also rich in dietary fibre, unsaturated fatty acids (around 60% of α-linolenic acid), and essential micronutrients, such as calcium, phosphorus, and magnesium, enhancing their overall nutritional value. Moreover, chia seeds are naturally gluten-free, similar to other crops such as quinoa, amaranth, and buckwheat, and can therefore be used in formulations for individuals with coeliac disease (Kulczyński et al., 2019). Overall, these characteristics make of chia seeds a plant-based source of complete protein with favourable techno-functional properties, suggesting their potential use in developing animal-based analogue food products. On the molecular level, chia seeds are characterised by several classes of storage protein, including 11S globulins (legumin-like proteins) and 7S globulins (vicilin-like proteins), which account the 60%–80% of the total protein content. In addition, chia seeds also contain smaller proportions of other protein fractions such as albumins, glutelins, prolamins, lipid transfer proteins (LTPs), and insoluble proteins (Sandoval-Oliveros and Paredes-López, 2013). Global production of chia seeds and their use as a food ingredient has grown significantly in recent years, thanks to their health benefits and growing consumer demand for protein. The potential of chia protein isolates and concentrates has been studied mainly using conventional extraction methods (e.g., dry or wet), among which alkaline one is the most commonly used technique (Khushairay et al., 2023). However, its impact on protein quality can vary depending on processing conditions (pH, temperature, and ionic strength) (Wang et al., 2023). Some studies report that chia seed protein concentrates and isolates can achieve a protein purity of approximately 60% and 90% respectively (López et al., 2018; Dirr et al., 2025).

Although chia seeds are generally recognised as safe for human consumption, there is still a potential risk of allergic reactions in susceptible individuals, so they must be carefully evaluated, especially when chia proteins are used in new food products. In the European Union (EU), chia seeds (*S. hispanica*) are classified as Novel food under Regulation (EU) 2015/2283 (European Parliament and of the Council and repealing Regulation; EFSA Panel on Nutrition et al., 2019a). The European Food Safety Authority (EFSA) requires all novel foods, especially proteins that may cause IgE-mediated allergic reactions, to undergo a comprehensive safety assessment (EFSA Panel on Dietetic Products and Nutrition and Allergies NDA, 2014). Concerns about the allergenic potential of chia proteins were first raised in earlier EFSA opinions. The EFSA Panel found evidence of cross-reactivity between chia proteins and sera from individuals allergic to sesame (*Sesamum indicum*), indicating the presence of structural similarities between these seeds. This was based on *in vitro* IgE-binding tests and skin prick tests (SPTs) (Opinion of the Scientific Panel; European Food Safety Authority (EFSA), 2009). Notably, sesame has recently been added to the list of the ‘Big Eight’ allergenic foods, due to its increasing prevalence and the severity of allergic reactions observed in sensitised individuals (Jiang et al., 2025). Although these early studies did not demonstrate any clinically confirmed allergic reactions, therefore the EFSA Panel concluded that cross-reactivity between chia and other seed allergens could not be ruled out. They also highlighted the lack of validated models or sufficient clinical data with which to predict the intrinsic allergenicity of chia proteins (European Food Safety Authority EFSA, 2009). More recently, EFSA’s NDA Panel (EFSA Panel on Nutrition et al., 2019a) reaffirmed that the manufacturing processes used to obtain chia-derived ingredients, such as protein powder, are not expected to alter their allergenic potential. However, limited clinical evidence has reported IgE-mediated reactions, including anaphylaxis and cutaneous symptoms, following chia consumption (Tomas-Pérez et al., 2018; Regula et al., 2023). Several allergenic proteins have been characterised in sesame (*S. indicum*), including 7S vicilin-like globulins (Ses i 3) and 11S legumin-like globulins (Ses i 6 and Ses i 7) (Jiang et al., 2025; Villa et al., 2024). Although chia seeds proteins have not yet been characterised at molecular level, previous studies have suggested that it contains homologous storage protein classes to those of sesame (Sandoval-Oliveros and Paredes-López, 2013). Such protein homologies could therefore explain the potential cross-reactivity observed between chia and other oilseed allergens, such as sesame.

Consequently, although chia seeds are a promising source of plant proteins, there is still limited experimental evidence in the current literature regarding their allergenic assessment, in particular at the molecular level. Therefore, to ensure its safe inclusion as a novel food, both the protein quality and allergenic potential of chia seeds require evaluation through molecular and immunological assessment. In this work, a protein extract compared with two raw reference and standardised samples from partial defatted chia flour were analysed to achieve a comprehensive molecular characterization and assess their potential allergenic risk. Thus, the main goal of this study was to establish a methodological framework that can be applied to novel protein sources to assess their allergenic potential and cross-reactivity risk with other already know allergens. This approach aimed to support a more reliable allergenicity assessment of novel food ingredients, in line with current EFSA recommendations combining molecular, *in silico*, and *in vitro* analyses (Mills et al., 2024).

## 2 Materials and methods

### 2.1 Sample description

In this work, three different samples of chia seeds (*S. hispanica*) were analysed. The description of the samples is given in Table 1. Chia raw material (CRM), chia standardised raw material (CSM), and chia protein concentrate (CPC) were analysed. All raw materials were purchased as partial defatted flour (PD) using a twin screw press (Olmühle Solling, Germany). CRM is a single batch of PD chia flour used as native reference material, while CSM consist of multiple batches of PD chia flour that has been standardised for macronutrient content (protein, fat and carbohydrates) to ensure uniformity between batches prior to protein extraction and functional testing. CPC, in particular, was a lyophilized concentrate obtained by alkaline precipitation/isoelectric point, pH 10 for solubilisation and pH 4 for precipitation. Samples were provided by the Hochschule Weihenstephan-Triesdorf (HSWT, Germany). Further details on the samples treatments and production can be found in a previous work (Dirr et al., 2025).

**TABLE 1:** Samples description.

Code	Sample	Description	Pre-treatment
CRM	Chia raw material	Raw material	Grinding
CSM	Chia standardised raw material	Raw material with standardized macronutrients content	Grinding
CPC	Chia protein concentrate	Extracted proteins with alkaline extraction (pH 10) and isoelectric point precipitation (pH 4), lyophilized powder	Grinding

### 2.2 Nutritional evaluation of proteins

#### 2.2.1 Protein content

The protein content was evaluated by determining the total nitrogen content using the Kjeldahl procedure, in accordance with the guidelines set out in European Regulation (EC) No 152/2009 (COMMISSION REGULATION, 2009). Digestion and distillation were performed using a DKL thermal digestion unit and a UDK 139 semi-automatic distillation system (VELP Scientifica, Usmate Velate, MB, Italy). The final protein concentration was obtained by multiplying the measured nitrogen content by the nitrogen-to-protein conversion factor calculated through amino acid analysis (5.42).

#### 2.2.2 Amino acid profile

The total amino acid composition was determined using the method described by Cutroneo et al. (2024). Briefly, the samples were subjected to acid hydrolysis, including a preliminary oxidation step with performic acid, to enable the accurate quantification of sulphur-containing amino acids. Norleucine was used as the internal standard in both procedures. The tryptophan content was determined through alkaline hydrolysis with α-methyl-tryptophan acting as the internal standard. Samples from acid hydrolysis and calibration curves were derivatized using the AccQ-Fluor reagent kit (Waters, Milford, MA, United States). All samples were analysed using an UHPLC ACQUITY system coupled with an ACQUITY SQ ESI-MS system (Waters, Milford, MA, United States). Data acquisition and processing was performed using MassLynx V4.0 software (Waters, Milford, MA, United States).

#### 2.2.3 Conversion factor estimation

The specific conversion factor (CF) was calculated using the amino acid composition by summing together the nitrogen contributions of each amino acid according to the following equation:

$$CF=\frac{100}{\%\;nitrogen}$$

#### 2.2.4 Amino acid score determination

The amino acidic score (AAS) for each essential amino acid was calculated according to the procedure described by Cutroneo et al. (2024). The AAS was defined as the ratio of the content of a given essential amino acid in the sample to the corresponding amino acid requirement pattern, both expressed in milligrams per gram of protein. The score for each sample was computed with respect to two population groups—children (3–18 years) and adults (>18 years) — as established by the FAO/WHO (FAQ, 2013).

### 2.3 Gel electrophoresis analysis

The sodium dodecyl sulfate-polyacrylamide gel electrophoresis (SDS-PAGE) analysis was performed according to the method described in our previous work (Calcinai et al., 2025), with slight modifications. In brief, the protein fraction of each samples (50 mg) was first extracted using 1.5 mL of extraction buffer (4 M urea, 100 mM ammonium bicarbonate (NH_4_HCO_3_), and 5 mM dithiothreitol (DTT)), and then quantified using a Qubit Fluorometer™ (Invitrogen, Carlsbad, CA, United States). Then, extracts containing 40 μg of protein were mixed with water, sample buffer 4× and reducing agent 20× (Bio-Rad, Hercules, CA, United States) and heated at 95 °C for 5 min. A molecular weight marker (Precision Plus Protein™ Prestained Standard) (Bio-Rad, Hercules, CA, United States) was used for estimating the apparent molecular weight (MW) of the separated protein bands. Protein separation was performed on a 10% Bis-Tris Criterion™ XT precast gel (Bio-Rad, Hercules, CA, United States), using a PowerPac power supply at a constant voltage of 150 V for approximately 45 min. The gel were stained with a Coomassie Brilliant Blue solution and subsequently destained. Finally, the gels were visualised using a GS-800™ calibrated densitometer (Bio-Rad, Hercules, CA, United States).

### 2.4 Peptide analysis

Prior to peptide analysis, the chia protein concentrate (CPC) sample was treated through an in-solution tryptic digestion procedure, according to the method described in literature (Cutroneo et al., 2024). Briefly, after protein extraction with buffer (4 M urea, 100 mM NH_4_HCO_3_, 5 mM DTT), proteins were subjected to tryptic digestion (trypsin from porcine pancreas - lyophilised powder, 1,000–2,000 BAEE units/mg solid - Sigma-Aldrich, St. Louis, MO, United States) after sequential reduction and alkylation. Then, samples were analysed in high-resolution mass spectrometry (HR-MS). High-resolution mass spectrometry (HRMS) analysis was performed using an Acquity I-Class UHPLC system (Waters, Milford, MA, United States) coupled to a Vion IMS QTof mass spectrometer (Waters, Milford, MA, United States), as described by Cutroneo et al., (2024) (Cutroneo et al., 2024). The data were acquired and processed using UNIFI software (Waters, Milford, MA, United States) with the same parameters as previously reported in the literature (Cutroneo et al., 2024). The data processing analysis was performed based on the sample protein composition determined with SDS-page analysis using the UniProt (https://www.uniprot.org/) (Uniprot, 2025) codes retrieved from sesame (*S. indicum*) proteins. The UniProt codes for the expected component list for each sample are listed in Table 2. Furthermore, the sequence coverage (%) between the peptides identified in the CPC sample and the *S. indicum* reference proteins was assessed using the Protein Coverage Summarizer (PCS) tool (http://omics.pnl.gov/software/ProteinCoverageSummarizer.php) (Protein coverage summarizer, 2025).

**TABLE 2:** UniProt (https://www.uniprot.org/) codes for the expected component listed for each sample.

Protein name	Uniprot code	Organism	Allergen name	References
2S seed storage protein 1	Q9XHP1	*Sesamum indicum*	Ses i 2	Wolff et al. (2003), Ma et al. (2020)
2S albumin	Q9AUD1	*Sesamum indicum*	Ses i 1	Wolff et al. (2003), Ma et al. (2020)
7S globulin	Q9AUD0	*Sesamum indicum*	Ses i 3	Jiang et al. (2025), Ma et al. (2020), Orruño and Morgan (2007)
11S globulin	Q9AUD2	*Sesamum indicum*	Ses i 7	Ma et al. (2020)
11S globulin seed storage protein 2	Q9XHP0	*Sesamum indicum*	Ses i 6	Ma et al. (2020), Wallowitz et al. (2007)
Oleosin H1	Q9FUJ9	*Sesamum indicum*	Ses i 4	Ma et al. (2020), Leduc et al. (2006)
Oleosin L	Q9XHP2	*Sesamum indicum*	Ses i 5	Ma et al. (2020), Leduc et al. (2006)

### 2.5 Allergenicity assessment

#### 2.5.1 Peptides conservancy analysis

Considering the limited data available on the allergenicity risk assessment of chia seeds, a comparative *in silico* analysis was conducted to examine potential sequence identity and cross-reactivity with already well-characterised sesame (*S. indicum*) allergens. Experimentally validated IgE-binding linear epitopes from *S. indicum* were retrieved from the Immune Epitope Database (IEDB) (https://www.iedb.org/) (IEDB, 2025). To explore conserved regions potentially involved in IgE cross-reactivity, identified chia seed peptides (Section 2.4), sesame allergenic proteins and IEDB-retrieved epitopes were subjected to multiple sequence alignment (MSA) using Clustal Omega (https://www.ebi.ac.uk/jdispatcher/msa/clustalo) (Katoh, 2021; Clustal Omega, 2025). The resulting alignments were graphically visualised and annotated using Jalview tool (https://www.jalview.org/) (Clamp et al., 2004; Jalview, 2025). For this analysis, only peptides with a minimum length of 6 amino acids were considered, as recommended by guidelines of the Codex Alimentarius (Ladics, 2008). The IEDB database and bioinformatic tools were last consulted in October 2025.

#### 2.5.2 *In vitro* allergenicity assays

Following the *in silico* assessment, *in vitro* immunoblotting assays were performed on chia seed extracts to evaluate their ability to bind to specific IgE antibodies in sera of allergic patients. The human *sera* used were provided by the Immunology Institute of the Santa Chiara Hospital in Pisa (Italy) and came from 2 patients allergic to sesame (sera code A and B). In addition, 1 serum from a non-allergic individual was included as a negative control (serum code C–no ImmunoCAP performed for sesame). An additional negative control was also performed with the sole use of the secondary antibody. The experimental protocol was approved by the Ethical Committee of the Pisa University Hospital (Approval No 19008/2021). Determination of allergen-specific IgE levels was performed at the same institute using the ImmunoCAP system. All specifics of the human sera used are reported in Supplementary Table S1. This analysis was performed according to the method described in our previous work (Calcinai et al., 2025), with some modifications. The primary hybridization was performed using human *sera* in a 1:5,000 ratio. The membrane was incubated at room temperature for 2 h under mild agitation (30 rpm). The secondary hybridisation was performed using an anti-human IgE (ε-chain specific) peroxidase antibody produced in goat (Sigma-Aldrich, St. Louis, MO, United States) (1:10,000 dilution) for 2 h at room temperature under mild agitation (30 rpm). Immunodetection has been carried out with the ChemiDoc MP Imaging System and analysed with Image Lab software (Bio-Rad).

### 2.6 Statistical analysis

Statistical analyses were carried out using the Statistical Package for the Social Sciences (IBM SPSS Statistics, version 26.0, IBM Corporation, Chicago, IL, United States), setting the significance threshold at ρ < 0.05. First, data distribution was assessed for normality, and then the appropriate parametric or non-parametric test was applied. The specific statistical test used is indicated in each table and figure caption.

## 3 Results

### 3.1 Nutritional evaluation of proteins

#### 3.1.1 Protein quantification

The protein content was determined for the three chia samples: CRM, CSM, and CPC. The results of the protein content analysis are reported in Table 3 as percentage values (on dry weight). Protein content was estimated from nitrogen determination by the Kjeldahl method and compared with the sum of total amino acid. For the conversion from nitrogen to protein, a specific conversion factor of 5.42 ± 0.05 – estimated from the amino acid profile of our chia samples–was used. The protein content ranged from the 26.1% in CRM and 25.6% in CSM, to the 56.6% in CPC, with statistically significant differences between the samples (ρ < 0.05). The sum of amino acids (AAs) followed the same trend, with respective values of 22.2, 22.4, and 51.8%.

**TABLE 3:** Total protein content of chia seed samples–chia raw material (CRM), chia standardized raw material (CSM), and chia protein concentrate (CPC) – determined with Kjeldahl method and as sum of total amino acids expressed as average (%) ± standard deviation.

Sample	Protein content (%)	Sum of AA (%)
CRM	26.1 ± 0.10^b^	22.4 ± 1.29^b^
CSM	25.6 ± 0.16^c^	22.2 ± 1.57^b^
CPC	56.6 ± 0.30^a^	51.8 ± 3.55^a^

Equal letters in columns correspond to values that are not significantly different (one-way ANOVA; Tukey’s test, ρ < 0.05).

#### 3.1.2 Amino acids profile

The amino acid composition of the chia samples is reported in Supplementary Figure S1. Glutamic acid was the dominant amino acid across all samples, followed by arginine, aspartic acid, and leucine. Conversely, sulphur-containing amino acids (methionine and cysteine) and tryptophan were found at the lowest levels. Statistical analysis revealed significant differences (ρ < 0.05) in the contents of several amino acids between the samples. In detail, the profile of CRM and CSM samples resulted completely comparable, while CPC showed variations in the content of some amino acids after alkaline extraction. An increase was, indeed, noted in lysine and tyrosine contents, while other amino acids (such as ala, cys, gly, ile, leu, thr, and val) decreased after extraction.

Going more into details of the amino acid quality, the sum of essential amino acids (EAAs) was investigated, and results are reported in Supplementary Figure S2. This value is around 437 mg/g of protein for all the chia samples, and no statistically significant differences were observed between them (ρ < 0.05). Aside from differences, all samples met and exceeded the FAO/WHO (2011) requirements for adults (277 mg/g protein) and children (291 mg/g protein) (FAQ, 2013). Anyhow, none of the samples reached the 512 mg/g of protein of egg white (used as reference) (Cutroneo et al., 2025a).

More detailed differences were investigated with the Amino Acid Score (AAS), that was calculated to evaluate the protein quality of each sample, as shown in Table 4. The AAS values indicated that lysine was the limiting amino acid in all samples for both adults and children, followed by valine. For the egg white reference protein, tryptophan was the only amino acid with a score below 1. Across the chia samples, CPC exhibited the highest AAS values overall, suggesting a more balanced composition of essential amino acids compared to CRM and CSM.

**TABLE 4:** Amino acid score of chia samples–chia raw material (CRM), chia standardized raw material (CSM), and chia protein concentrate (CPC). Results are expressed as the ratio between the amino acid content of each sample and the FAO/WHO reference values (WHO, 2011).

Children (3–18 years)	His	Ile	Leu	Lys	SAA	AAA	Thr	Trp	Val
CRM	1.73	1.33	1.23	**1.10**	2.40	2.06	1.71	1.36	1.23
CSM	1.78	1.26	1.25	**1.03**	2.42	2.12	1.81	1.36	1.18
CPC	1.89	1.20	1.19	1.31	2.33	2.33	1.53	1.23	**1.04**
Adults	**His**	**Ile**	**Leu**	**Lys**	**SAA**	**AAA**	**Thr**	**Trp**	**Val**
CRM	1.85	1.33	1.27	**1.17**	2.50	2.23	1.86	1.49	1.27
CSM	1.90	1.26	1.30	**1.10**	2.53	2.29	1.96	1.50	1.21
CPC	2.01	1.20	1.23	1.40	2.44	2.51	1.66	1.35	**1.06**
Egg reference	**His**	**Ile**	**Leu**	**Lys**	**SAA**	**AAA**	**Thr**	**Trp**	**Val**
CRM	1.26	0.74	0.87	0.75	0.97	0.91	0.91	**0.53**	0.75
CSM	1.29	0.70	0.89	0.71	0.98	0.94	0.96	**0.53**	0.72
CPC	1.37	0.66	0.84	0.90	0.94	1.03	0.81	**0.48**	0.63

Values in bold: limiting amino acids; Value underlined: below 1;

SAA, sulphuric amino acids (methionine and cysteine); AAA, aliphatic amino acids (phenylalanine and tyrosine).

### 3.2 Protein profile characterisation

**FIGURE 1:**
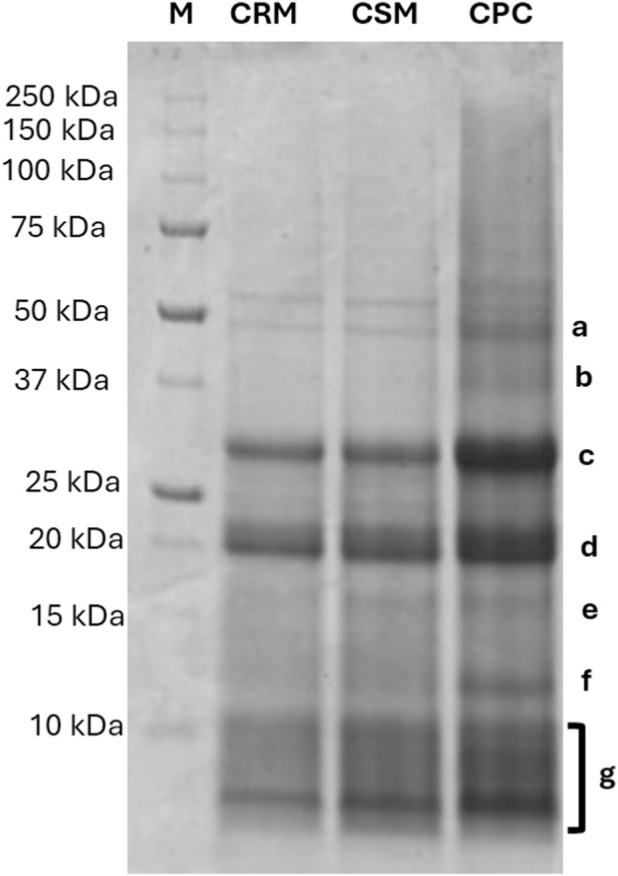
SDS-PAGE of the chia samples. Lane 1: chia raw material (CRM); lane 2: chia standardised material (CSM); lane 3: chia protein concentrate (CPC); M: molecular weight markers (kDa).

The protein profiles of samples (CRM, CSM, and CPC) were analysed by SDS-PAGE under reducing conditions. The electrophoresis gel is shown in Figure 1.

Multiple protein bands with distinct molecular weights ranging from approximately 50 to 10 kDa were observed. The identification of proteins was performed in comparison with the literature. Based on the literature, the chia seed proteins identified correspond to homologous storage proteins from sesame (*S. indicum)*. Previous studies identified sesame proteins using high-resolution mass spectrometry (HR-MS) and then matched them by homology with *S. hispanica* proteins, as the latter lacks a sequenced genome (Sandoval-Oliveros and Paredes-López, 2013). The tentative attribution of protein bands, with corresponding UniProt accession numbers, is reported in Table 5. The most intense band (approximately at 50 kDa), corresponded to the 11S globulin precursor, which is a major seed storage protein (Sandoval-Oliveros and Paredes-López, 2013; Ma et al., 2020). The band at approximately 40 kDa, was identified as 7S globulin (Sandoval-Oliveros and Paredes-López, 2013; Orruño and Morgan, 2007). Two additional bands at 30 and 20 kDa, respectively, were consistent with the acidic and basic chains of 11S globulin, as reported in literature (Sandoval-Oliveros and Paredes-López, 2013; Wallowitz et al., 2007). At lower molecular weights, the protein band at around 16 kDa was identified as oleosin H1 and oleosins L (Leduc et al., 2006). Finally, proteins in the range between 14 and 8 kDa (bands g and h) were identified as 2S albumin (Wolff et al., 2003).

**TABLE 5:** Protein identified in the SDS-PAGE of the three chia seeds (*Salvia hispanica*) samples by comparison with current literature.

Band code	Theoretical MW (kDa)	Protein	Uniprot code^a^	Protein existence^b^	Species	References
a	50	11S globulin precursor	Q9AUD2	2	*Sesamum indicum*	Sandoval-Oliveros and Paredes-López (2013), Ma et al. (2020)
b	40	7S globulin	Q9AUD0	2	*Sesamum indicum*	Sandoval-Oliveros and Paredes-López (2013), Jiang et al. (2025), Orruño and Morgan (2007)
c	30	11S globulin (acidic chain)	Q9XHP0	1	*Sesamum indicum*	Sandoval-Oliveros and Paredes-López (2013), Wallowitz et al. (2007)
d	20	11S globulin (basic chain)	Q9XHP0	1	*Sesamum indicum*	Sandoval-Oliveros and Paredes-López (2013), Wallowitz et al. (2007)
e	16	Oleosin H1	Q9FUJ9	1	*Sesamum indicum*	Leduc et al. (2006), Rodríguez et al. (2024)
		Oleosin L	Q9XHP2	1	*Sesamum indicum*	Leduc et al. (2006), Rodríguez et al. (2024)
f	12	2S albumin	Q9AUD1	2	*Sesamum indicum*	Sandoval-Oliveros and Paredes-López (2013), Wang et al. (2023), Wolff et al. (2003)
g	8		Q9XHP1	1	*Sesamum indicum*	Sandoval-Oliveros and Paredes-López (2013), Wang et al. (2023), Wolff et al. (2003)

^a^ Protein identification was based on homology with *Sesamum indicum* proteins reported in the literature.

^b^ Indication of the type of evidence that supports the existence of the protein: 1. Experimental evidence at protein level; 2. Experimental evidence at transcript level; 3. Protein inferred from homology; 4. Protein predicted; 5. Protein uncertain. Evaluated with Uniprot (https://www.uniprot.org/).

### 3.3 Peptide analysis following in solution tryptic digestion

In-solution tryptic digestion of chia protein concentrate (CPC), followed by high-resolution mass spectrometry (HR-MS) analysis, was used to identify several peptides corresponding to the main proteins of *S. indicum* (Table 2; Section 2.4) that can be present also in chia proteins due to similarity. The identified proteins and their respective UniProt accession codes, number of peptides, peptide length and sequence coverage (%) are summarised in Table 6. The complete list of peptides identified by HR-MS is provided in Supplementary Table S2. The 7S globulin among the storage proteins showed the highest peptide number (EU, 2011) and coverage (78%), while the 11S globulin precursor (32 peptides) and the 11S globulin (28 peptides) showed coverage values of 64% and 63%, respectively. Oleosin H1 exhibited a coverage of 80% over a number of peptides of 7, whereas oleosin L resulted in 3 identified peptides with a limited sequence coverage (12%). For the 2S albumins, coverage values ranged from 72% to 85%. To the best of our knowledge, coverages observed in this study are much higher to the ones observed in literature (Sandoval-Oliveros and Paredes-López, 2013), highlighting the robustness of the methodology used. Graphical elucidation of the coverages with peptide identified sequences alignment is reported in Supplementary Figures S3–S9. The mapping of peptides from in-solution digestion in high resolution mass spectrometry, which was observed to be in accordance with the tentative protein identification, confirms the identification previously proposed.

**TABLE 6:** Peptide identification of chia seeds protein concentrate by in solution tryptic digestion coupled with HR-MS analysis (Vion IMS Qtof) with sesame (*Sesamum indicum*) protein sequences.

Protein	Uniprot code	N° peptides	Average lenght	Coverage (%)^a^
11S globulin precursor	Q9AUD2	32	19	64
7S globulin	Q9AUD0	44	22	78
11S globulin	Q9XHP0	28	20	63
Oleosin H1	Q9FUJ9	7	22	80
Oleosin L	Q9XHP2	3	9	12
2S albumin	Q9XHP1	15	24	72
2S albumin	Q9AUD1	18	22	85

^a^ Sequence coverage was calculated using the Protein Coverage Summarizer tool (http://omics.pnl.gov/software/ProteinCoverageSummarizer.php) and graphically elucidated in Supplementary Figures S3–S9 of the Supplementary Material.

### 3.4 Allergenicity assessment

#### 3.4.1 Peptide conservancy analysis with sesame allergens

Multiple sequence alignments were performed between identified peptides of chia seed protein concentrate (CPC) and experimentally validated linear IgE-binding epitopes of *S. indicum* allergens, namely, Ses i 2 (Q9XHP1) (Wolff et al., 2003) and Ses i 5 (Q9XHP2) (Yu et al., 2025). The experimentally validated epitopes retrieved from IEDB (https://www.iedb.org/) and used as references in this analysis are listed in Supplementary Material Supplementary Table S3 for Ses i 2 (Q9XHP1) and Ses i 5 (Q9XHP2), respectively. These two proteins were selected because they are currently the only sesame allergens with experimentally validated linear epitopes available in IEDB.

**FIGURE 2:**
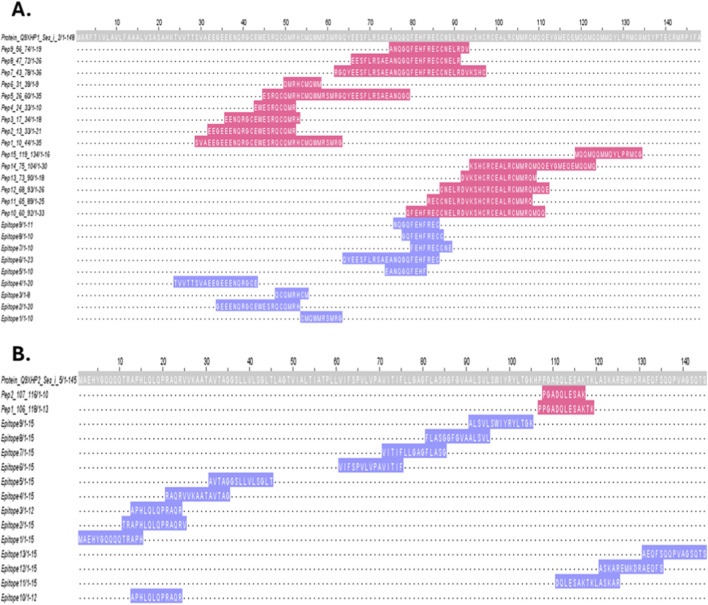
Multiple sequence alignments were performed between HR-MS identified peptides of chia seeds and *Sesamum indicum* allergens. **(A)** Alignment of chia-identified peptides with Ses i 2 (UniProt ID: Q9XHP1); **(B)** Alignment of chia-identified peptides with Ses i 5 (UniProt ID: Q9XHP2). Grey bars represent full-length sesame reference protein sequences, while pink regions indicate peptides identified by HR-MS in chia seed protein concentrate. Light blue regions correspond to IgE-binding linear epitopes that have been experimentally validated and retrieved from the Immune Epitope Database (IEDB, https://www.iedb.org/). The alignments were generated using Clustal Omega (https://www.ebi.ac.uk/jdispatcher/msa/clustalo) and visualised with Jalview (https://www.jalview.org/).

The graphical representation of the multiple sequence alignments generated with Jalview (https://www.jalview.org/) is shown in Figure 2A for the 2S albumin, Ses i 2 (Q9XHP1) and in Figure 2B for the Oleosin L, Ses i 5 (Q9XHP2). The grey bars represent the full-length amino acid sequences of the *S. indicum* reference proteins, the pink regions correspond to peptides of chia seed proteins identified in this work by HR-MS, and the light blue grey segments indicate the experimentally validated epitopes retrieved from the IEDB. For Ses i 2 (Q9XHP1) (Figure 2A), several regions of overlap were observed between chia-derived peptides and known sesame epitopes, primarily within the N-terminal region of the allergen, where 8–12 amino acid residues were found to be identical. In contrast, no regions of overlap were found between chia peptides and the reported epitopes of Ses i 5 (Q9XHP2), indicating an absence of shared linear sequence motifs in this allergen.

#### 3.4.2 IgE-binding capacity of chia seed proteins with human sera allergic to sesame

**FIGURE 3:**
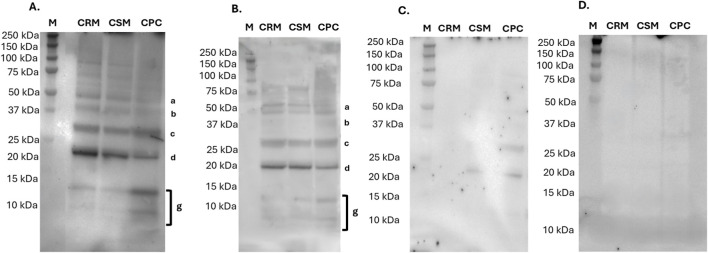
Immunoblotting of chia samples–chia raw material (CRM), chia standardized raw material (CSM), and chia protein concentrate (CPC) – with sera from patient allergic to sesame: serum A **(A)** and serum B **(B)**, negative control **(C)** and secondary antibody only control **(D)** –, M, molecular weight marker. Putative band attribution: a) 11S globulin precursor; band b) 7S globulin; band c) 11S globulin acidic chain; band d) 11S globulin basic chain; band g) 2S albumin.

Immunoblotting assays were performed using two human sera from patients with a sesame (*S. indicum*) allergy (Section 2.5.3) to evaluate the IgE-binding reactivity towards the three chia seed protein samples. The immunoblotting assays are shown in Figure 3.

Serum A (Figure 3A) displayed IgE recognition of several protein bands, with the most intense signals observed at approximately 50 (band a), 40 (band b), 30 (band c), and 20 (band d) kDa. These corresponded, respectively, to the tentative sequences identified as 11S globulin precursor, the 7S globulin and the acidic and basic chains of the 11S globulin. Additional faint reactivity was detected between 12 and 8 kDa (band g), consistent with 2S albumin protein. Furthermore, a few reactive bands above 50 kDa were visible in immunoblotting but not detected in the corresponding SDS-PAGE profiles (Section 3.2; Figure 1), likely due to their low abundance in the samples. On the other hand, serum B (Figure 3B) exhibited weaker IgE-binding intensity, only against the tentatively attributed 11S globulin precursor (band a, 50 kDa), 7S globulin (band b, 40 kDa) and acidic and basic chains of the 11S globulin (bands c and d, 30 and 20 kDa). For both sera, a stronger reactivity of proteins with molecular weight ≥20 kDa was noted in the raw materials (CRM and CSM), while lower IgE-binding intensity was observed in the CPC. On the contrary, in sera A, a higher detected reactivity was noted in lower molecular weight bands (g and h) for the CPC than CRM and CSM samples. No specific IgE-binding protein bands were observed in the negative control serum (Figure 3C). However, there is no documented absence of sesame-specific IgE, therefore subclinical sensitization cannot be excluded. Furthermore, no IgE-binding protein bands were observed in the secondary antibody only control (Figure 3D). This allows to exclude non-specific binding to chia protein of the anti-human IgE antibody used in this study.

## 4 Discussion

The results obtained provide a comprehensive insight on chia seed (*S. hispanica*) proteins, with the novelty of a complete characterization at a molecular level regarding their nutritional quality, protein class identification, theorized peptide mapping, and evaluation of the allergenic potential. Results are pivotal to have a conscious and safe use, given their increasing employ in the food industry as recent novel food (EFSA Panel on Nutrition et al., 2019a). The results contribute to fill the gap in essential knowledge regarding the protein adequacy of chia seeds and their potential cross-reactivity with well-characterised allergens found in other oilseeds, such as sesame (*S. indicum*) (EFSA Panel on Nutrition et al., 2019b).

### 4.1 Protein quality

The protein quality was investigated to confirm whether the extraction affected or not the intrinsic quality of the raw material. As well known, indeed, chia is a nutritious oilseed with a good protein content and that brings all essential amino acids (Kulczyński et al., 2019), this leads it to be a well-balanced and complete source of proteins. On the other hand, when extracted proteins can vary their functionality, composition, and nutritional quality due to the treatment applied (Wang et al., 2023). In our sample case, the protein extraction–performed with a defatting pre-process and alkaline solubilization with pI precipitation (Dirr et al., 2025) – was effecting in recovery a good amount of proteins from the raw materials, producing a protein concentrate with more than doubled content of proteins in comparison with the raw materials. Its protein content was observed to be around 56%. The protein content was exactly determined with the use of a specific conversion factor (5.42 ± 0.05 as average of the CF of both raw materials and protein concentrate) estimated with the amino acid composition. The use of accurate nitrogen to protein conversion factors is essential to estimate the protein content of a food/ingredient (Cutroneo et al., 2025b). Indeed, the use of general conversion factors as 6.25 (as indicated by the Regulation (EU) No 1169/2011 on nutrition facts (EU, 2011) of literature references (i.e. 5.7 (Ayerza, 2009)) can bring to an overestimation of the protein content. This leads to the incorrect calculation when converting with animal-based proteins and/or when it is used as ingredient with the goal to formulate nutritious products (Cutroneo et al., 2025b; Mariotti et al., 2008).

More important in this optic is the AA composition and, in particular, the EAA and AAS. The two raw materials showed complete comparability between each other with minimal differences observed in the extracted sample showing that the extraction treatment slightly affected the AA profile but not drastically. The AA profile of the chia samples was in accordance with the literature. Studies highlighted, indeed, its high content in both essential–lysine (0.97 g/100 g), leucine (1.10 g/100 g), valine (0.91 g/100 g), and phenylalanine (0.92 g/100 g) – and non-essential–glutamic acid (3.50 g/100 g), aspartic acid (1.50 g/100 g), and glycine (0.80 g/100 g) – amino acids (Kulczyński et al., 2019). When moving to the EAAs, the sum was more than sufficient to fulfil the requirements set by FAO/WHO for children and adults (FAQ, 2013). This indicates the adequacy of this protein source to be used as a good source of proteins. Anyhow, when compared to the egg white protein values (Cutroneo et al., 2023) – which is considered the complete protein (Yu and Fukagawa, 2020) – this is not true. For this reason, to give a deeper understanding in the protein quality, the AAS was also observed. Results are concordant with the AAs profile and EAA. The AAS of chia samples is, indeed, not comparable with the egg white reference protein (Yu and Fukagawa, 2020). On the other hand, when comparing with the scoring patterns given by FAO/WHO (FAQ, 2013), the AAS of chia samples is more than enough to fulfil the requirements for both children and adults population groups, with scores all above 1. The limiting amino acid was observed to be lysine for both CRM and CSM, while it is valine for CPC. This result is concordant with the AA profile, where lysine increased after protein extraction. The observed scores, all above 1, confirmed the adequacy of chia for the human nutrition and are in accordance with what retrieved from the literature (Wang et al., 2023).

### 4.2 Protein molecular characterization

If on one hand the protein quality of chia is already known, on the other hand its protein composition and characterization at a molecular level lacks (Sandoval-Oliveros and Paredes-López, 2013; EFSA Panel on Nutrition et al., 2019b). For this reason, protein fraction of chia was characterized by comparison with sesame. Starting from well-established similarities between chia and sesame proteins (Sandoval-Oliveros and Paredes-López, 2013) proteins were identified, and their identification was confirmed *via* HR-MS. Moreover, the theoretical sequence of peptides homologous with sesame proteins was reported.

#### 4.2.1 Protein identification

The SDS-PAGE profiles of all chia seed samples showed similar patterns, with, as expected, a higher intensity of the band for the protein extract (CPC). This indicates that the extraction process did not alter the protein profile of the CPC (Figure 1; Section 3.2). The most prominent bands appeared at approximately 50 and 45 kDa, corresponding to 11S globulin precursors and 7S globulins, respectively. These proteins belong to the cupin superfamily and represent the main classes of seed storage proteins commonly found in plants (Costa et al., 2022). Additional bands at 30 and 20 kDa were assigned to the acidic and basic subunits of 11S globulins, respectively, based on literature data (Sandoval-Oliveros and Paredes-López, 2013). Minor bands observed between 17 and 15 kDa were attributed to oleosin proteins (Sandoval-Oliveros and Paredes-López, 2013; Leduc et al., 2006; Rodríguez et al., 2024), while those in the 12.5–8 kDa range were associated with 2S albumins (Sandoval-Oliveros and Paredes-López, 2013; Wang et al., 2023; Wolff et al., 2003). Tentative identified sequences agree with those reported by Sandoval-Oliveros and Paredes-López (2013), who found that the globulin fraction accounted for about 52% of the total protein in chia flour, consisting mainly of 11S and 7S storage proteins. More recently, Ziaeifar et al. (2025) confirmed that, although extraction conditions may affect protein integrity, the globulin and albumin fractions remain the predominant and functionally relevant proteins in chia seeds.

Since the chia seed genome has not yet been sequenced and no specific UniProt entries are available for this species, high-resolution mass spectrometry (HR-MS) was essential in this study. It enabled the molecular identification of chia seed peptides by homology with well-characterized proteins from sesame (*S. indicum*). According to previous studies (Sandoval-Oliveros and Paredes-López, 2013; Gupta et al., 2023), all proteins identified in *S. hispanica* showed homology with those from sesame, although sequence coverage was relatively low. This is typical of proteomic studies on species lacking full genomic data, where achieving high sequence matching is challenging (Sandoval-Oliveros and Paredes-López, 2013). This lack of molecular data for *S. hispanica* proteins represents a limitation in assessing the allergenic potential of chia seeds. The EFSA scientific opinion (2019) (EFSA Panel on Nutrition et al., 2019b) reported *in vitro* evidence of IgE cross-reactivity between chia seed proteins and serum from sesame-allergic patients, but confirmation at the molecular level is still lacking. In this context, the present study aims to provide new insights through a comprehensive proteomic identification of chia seed peptides based on their homology with known sesame proteins.

#### 4.2.2 Peptide analysis and sequence homology by HR-MS

Due to the absence of protein sequence data for *S. hispanica* in existing databases, an in-solution tryptic digestion followed by high-resolution mass spectrometry (HR-MS) analysis was carried out on the chia seed extracted proteins. Peptide identification was performed using *S. indicum* proteins as references, focusing on those corresponding to the major bands observed in the SDS-PAGE profile (Figure 1; Section 3.2). The peptides identified in chia were mapped onto the corresponding sesame protein sequences to determine sequence coverage (%), confirming the expected homology between the two species. As showed, high sequence coverages were obtained for the 11S globulin precursor (Q9AUD2) and the 7S globulin (Q9AUD0), supported by a high number of peptides and high coverages (>64%). The 11S globulin (Q9XHP0) was also well represented, with 28 peptides identified and a sequence coverage of 63%. Within the 2S albumin family, several proteins were identified, coverage values ranging from 85% to 57%. These findings confirm that chia and sesame proteins, both globulins and albumins, share a high degree of sequence homology, especially within the conserved structural regions typical of seed storage proteins belonging to the cupin superfamily (Sandoval-Oliveros and Paredes-López, 2013; Ziaeifar et al., 2025). Among the oleosins, oleosin H1 (Q9FUJ9) showed high coverage of 80% based on 7 peptides. Thanks to the HR-MS techniques combined with a deep knowledge of sesame it was possible to confirm the presence of the same classes of sesame storage proteins–globulins, albumins, and oleosins–in chia seeds. Nevertheless, the high coverage observed suggested sequence homologies that could contribute to potential cross-reactivity, as highlighted by the EFSA NDA Panel (2019b) for *S. hispanica* proteins. Furthermore, results–reporting sequence of theoretical peptides of chia homologous to sesame–are the first step towards a complete understanding of the chia protein sequences. Nevertheless, as already mentioned, the peptide coverages observed in this study substantially exceed those reported in literature (Sandoval-Oliveros and Paredes-López, 2013), underscoring the methodology’s robustness. High-resolution MS mapping of in-solution digestion peptides aligns with tentative protein identifications, confirming prior assignments.

### 4.3 Allergenicity assessment: *in silico* and *in vitro* combined approach

The combination of *in silico* and *in vitro* analyses provided complementary insights into the allergenic potential of chia (*S. hispanica*) proteins. The *in silico* approach enabled the prediction of potential IgE-binding regions through sequence comparison with known sesame (*S. indicum*) allergens, while *in vitro* immunoblotting validated these findings by testing actual IgE recognition using sera from sesame-allergic patients. This combination of strategy is consistent with current EFSA recommendations for the allergenicity assessment of novel proteins, which emphasize the importance of integrating bioinformatic prediction with *in vitro* validation (Mills et al., 2024). The *in silico* peptide conservancy analysis revealed sequence homology between chia seed peptides and the experimentally validated linear IgE-binding epitopes of sesame allergens, particularly Ses i 2 (2S albumin) (Figure 2A, Section 3.4.1) (Wolff et al., 2003). Thus, this conservancy supports the molecular basis for cross-reactivity that has been previously noted in clinical studies (Tomas-Pérez et al., 2018; EFSA Panel on Nutrition et al., 2019b). Notably, the *in silico* results were confirmed by our *in vitro* immunoblotting results (Figure 3; Section 3.4.2), in which sera from sesame-allergic patients showed IgE-binding reactivity to low molecular weight protein bands compatible with chia seeds 2S albumin proteins. These results confirmed that 2S albumins are among the main proteins responsible for cross-reactivity between chia seeds and sesame (Albunni et al., 2019). On the other hand, the absence of regions conserved with the Ses i 5 allergen (oleosin L) (Figure 2B, Section 3.4.1) is consistent with the results of the *in vitro* immunoblotting performed in this study, which did not show any IgE-binding to low molecular weight proteins corresponding to oleosins. *In vitro* IgE-binding tests were performed using sera provided by the Immunology Institute of the Santa Chiara Hospital in Pisa (Pisa, Italy), including sera from patients allergic to sesame and one serum from an individual with a diagnosed IgE-mediated allergy to milk proteins, used as a non-allergic control for sesame. Sesame-specific testing was not performed for the this serum, as no clinical evidence suggested allergy to sesame or other seeds and immunological tests were performed on the basis of clinical indications. The immunoblotting assays using sera from sesame-allergic patients showed also strong IgE-binding reactivity against higher molecular weight proteins, corresponding to 11S globulin precursor (Ses i 7), 7S globulin (Ses i 3), and 11S globulin subunits (Ses i 6). These findings reflected not only the high sequence coverage observed, but also the known immunodominance of these globulin classes in oilseeds allergy (Costa et al., 2022; Mills et al., 2003). Finally, reactive bands above 50 kDa were observed in the immunoblotting (Figure 3; Section 3.4.2) but were not visible in the corresponding SDS-PAGE profiles (Figure 1; Section 3.2). These bands can be attributed to globulin aggregates, as reported in the literature (Sandoval-Oliveros and Paredes-López, 2013). Such aggregates may have retained regions that can be recognized by IgE, which could explain the observed reactivity despite their low abundance. The variation in IgE-binding intensity among different patient sera reflects the diverse sensitization profiles, indicating that individual immune responses to allergens can differ significantly (Dramburg et al., 2023). In this context, serum A showed a higher IgE-binding intensity than serum B, further confirming individual variability in allergen recognition. Moreover, the CPC sample showed a generally stronger IgE-binding than the CRM and CSM ones. This fact may reflect the effect of protein concentration, which can increase the relative abundance of IgE binding sites, enhancing immune recognition (Foo and Mueller, 2021). Therefore, it is important to consider processing and extraction methods in the assessment of allergenicity, particularly for novel foods, as they may influence epitope exposure and thus allergic responses.

Indeed, the *in silico* and *in vitro* results obtained in this study indicate that the allergenic potential of chia seeds can be mainly associated with globulin and albumin proteins, which share high sequence homology and conserved epitopes with known sesame allergens. Observed results suggest the need to expand the number of sera studied in order to provide more robust conclusions. Anyhow, this study findings confirmed the presence of immunological similarities and suggest a potential heterologous allergenicity between chia seed and sesame proteins. Moreover, they suggests the possibility of cross-reactivity in individuals allergic to sesame and support the EFSA NDA Panel (2019) opinion (EFSA Panel on Nutrition et al., 2019b) on the importance of declaring the use of chia seeds as novel food ingredients, as well as the need for continued clinical and immunological evaluation.

## 5 Conclusion

This study provided a comprehensive molecular characterization of chia seed (*S. hispanica*) proteins, focusing on their quality and potential allergenicity evaluation as a novel food source. From a protein quality perspective, chia seeds proteins possessed a balanced amino acid composition and adequate amino acid scores, confirming their value as a high-quality plant-based protein source. The fulfilment of all requirements set by FAO/WHO make of chia a good substitute for animal proteins, even if not comparable with the egg white. Further studies are needed in this optic to confirm whether or not those values are kept also after digestion, estimating the bioavailability of proteins.

The protein quality is, anyhow, only one of the many factors to consider when a new source of proteins is considered. The current lack of comprehensive data on the allergenic risk of chia seeds proteins, highlights the need for a more in-depth allergenicity assessment before their inclusion in food formulations. To the best of our knowledge, this is the first study able to identify parts of chia protein sequences. The putative attributions, supported by the peptide mapping in high resolution mass spectrometry, are proposed as a first building block to the sequencing and therefore more robust identification of chia seeds proteins. Moreover, the novelty of the approach can be useful to evaluate the allergenic potential of several different protein sources.

The identification of theoretical peptides was not only essential to confirm homology with sesame proteins thanks to coverages, but it also gave the possibility to predict possible epitopes regions. These results combined with *in silico* and *in vitro* analyses revealed that the main allergenic potential of chia seeds is associated with globulin and albumin proteins, which shared high sequence homology and conserved IgE-binding epitopes with known sesame allergens. These results confirmed previous evidence. Moreover, these findings support the presence of immunological similarities and suggest a potential heterologous allergenicity between chia seed and sesame proteins. However, these results should be considered preliminary, as the *in vitro* immunoblotting tests were performed on a limited number of sera and were intended to provide proof of concept rather than clinical validation. To confirm the clinical relevance of the results, indeed, further studies needs to be performed on an higher number of sera with patients subjected to prick-to-prick tests, to confirm the hypothesized allergy to chia. The strong IgE-binding signals observed in the chia protein concentrate further emphasised that protein extraction methods could influence epitope exposure, thereby influencing immune recognition.

Overall, this study contributed to the molecular-level understating of chia seeds proteins allergenicity and emphasised the importance of integrating proteomic and immunological data for the allergenicity assessment of novel food proteins. Further studies on larger samples of allergic individuals and specific clinical research will be necessary to confirm and increase the robustness of these findings. In line with current EFSA recommendations, a clear allergen labelling and further clinical studies are required to ensure the safe introduction of chia-based ingredients in food formulations suitable for sensitised consumers.

## Data Availability

All research data supporting the findings of this manuscript are available in accordance with PRIMA and Horizon 2020 open data requirements, and in line with Horizon Europe’s Open Science principle, ensuring that knowledge produced with EU funds is accessible and reusable. Following the Open Research Data (ORD) policy, this project has made all data FAIR (Findable, Accessible, Interoperable, Reusable). The datasets are freely accessible via the Zenodo repository at https://zenodo.org/communities/proximed/records. The data of this study are reported at doi 10.5281/zenodo.19402495.
