# Supplementary material for: Protein quality and allergenicity assessment of chia seeds (Salvia hispanica): a molecular perspective on novel food safety
Source: Front Toxicol. 2026 Apr 20;8:1735718. doi: 10.3389/ftox.2026.1735718 (PMC13135838; doi:10.3389/ftox.2026.1735718)
Supplement: Supplementary file 1 [file Supplementaryfile1.docx]

***Supplementary Material***

**1. Supplementary Tables**

**Table S1**. Detailed characteristics of human sera under study.

| **Serum code** | **Age** | **Sex** | **Immunocap Specific IgE** | | **After Chia Exposure** | | | **Other sensitizations** |
| --- | --- | --- | --- | --- | --- | --- | --- | --- |
|  |  |  | **Allergy** | **kUA/L^*^** | Symptoms | Time | Therapy needed |  |
| A | 55 | F | Sesame | 4.6 | Respiratory and  abdominal | 1 hour | Pharmacological therapy based on steroids and antihistamines; no adrenaline required | Foods: Legumes, nuts, seeds  and fruits.  Others: pollen |
| B | 56 | M | Sesame | 1.20 | Urticaria, angioedema, and widespread pruritus | 1 hour | Pharmacological therapy based on steroids and antihistamines; no adrenaline required | Food: fruits, wheat, and milk.  Others: olive pollen, house dust mites |
| C | 54 | F | Milk | 0.58 | No reported hypersensitivity reactions to sesame | - | - | Red meat |

^*^ Specific IgE values were determined by ImmunoCAP test; values ≥ 0.35 kUA/L were considered positive (1).

**Table S2**. List of peptides identified in chia seeds protein concentrate by in solution tryptic digestion coupled with HR-MS analysis (Vion IMS Qtof) with sesame (*Sesamum indicum*) protein sequences

| **Protein name** | **Accession**  **N Uniprot** | **Peptide N** | **Sequence start *** | **Sequence end *** | **Peptide sequence** | **Lenght (AA)** |
| --- | --- | --- | --- | --- | --- | --- |
| 11S globulin precursor | Q9AUD2 | 32 | 10 | 38 | WQDLQSQQQHKLQARTDCRVERLTAQEPT | 29 |
|  |  |  | 32 | 40 | LTAQEPTIR | 9 |
|  |  |  | 48 | 67 | TEFWDRNNQQFECAGVAAVR | 20 |
|  |  |  | 50 | 86 | FWDRNNQQFECAGVAAVRNVIQPRGLLLPHYNNAPQL | 37 |
|  |  |  | 51 | 71 | WDRNNQQFECAGVAAVRNVIQ | 21 |
|  |  |  | 52 | 60 | DRNNQQFEC | 9 |
|  |  |  | 59 | 82 | ECAGVAAVRNVIQPRGLLLPHYNN | 24 |
|  |  |  | 74 | 88 | GLLLPHYNNAPQLLY | 15 |
|  |  |  | 74 | 91 | GLLLPHYNNAPQLLYVVR | 18 |
|  |  |  | 98 | 116 | TVIPGCAETFERDTQPRQD | 19 |
|  |  |  | 100 | 108 | IPGCAETFE | 9 |
|  |  |  | 102 | 126 | GCAETFERDTQPRQDRRRRFMDRHQ | 25 |
|  |  |  | 112 | 115 | QPRQ | 4 |
|  |  |  | 125 | 132 | HQKVRQFR | 8 |
|  |  |  | 157 | 190 | TVALLDTGNAANQLDQTFRHFFLAGNPQGGRQSY | 34 |
|  |  |  | 179 | 199 | LAGNPQGGRQSYFGRPQTEKQ | 21 |
|  |  |  | 180 | 204 | AGNPQGGRQSYFGRPQTEKQQGETK | 25 |
|  |  |  | 184 | 217 | QGGRQSYFGRPQTEKQQGETKNIFNGFDDEILAD | 34 |
|  |  |  | 187 | 198 | RQSYFGRPQTEK | 12 |
|  |  |  | 196 | 206 | TEKQQGETKNI | 11 |
|  |  |  | 221 | 227 | VDVQTAR | 7 |
|  |  |  | 252 | 288 | EEEEERWERDPYSGANGLEETLCTAKLRENLDEPARA | 37 |
|  |  |  | 259 | 287 | ERDPYSGANGLEETLCTAKLRENLDEPAR | 29 |
|  |  |  | 274 | 299 | CTAKLRENLDEPARADVYNPHGGRIS | 26 |
|  |  |  | 326 | 340 | LVAPHWNLNAHSIIY | 15 |
|  |  |  | 328 | 337 | APHWNLNAHS | 10 |
|  |  |  | 393 | 411 | KTNDNAMTSQLAGRLSAIR | 19 |
|  |  |  | 409 | 430 | AIRAMPEEVVMTAYQVSRDEAR | 22 |
|  |  |  | 421 | 428 | AYQVSRDE | 8 |
|  |  |  | 431 | 454 | RLKYNREESRVFSSTSRYSWPRSS | 24 |
|  |  |  | 435 | 447 | NREESRVFSSTSR | 13 |
|  |  |  | 437 | 453 | EESRVFSSTSRYSWPRS | 17 |
| 7S globulin | Q9AUD0 | 44 | 9 | 14 | QCKHQC | 6 |
|  |  |  | 13 | 30 | QCKAQQQISKEQKEACIQ | 18 |
|  |  |  | 21 | 61 | SKEQKEACIQACKEYIRQKHQGEHGRGGGDILEEEVWNRKS | 41 |
|  |  |  | 28 | 54 | CIQACKEYIRQKHQGEHGRGGGDILEE | 27 |
|  |  |  | 29 | 33 | IQACK | 5 |
|  |  |  | 30 | 65 | QACKEYIRQKHQGEHGRGGGDILEEEVWNRKSPIER | 36 |
|  |  |  | 42 | 57 | GEHGRGGGDILEEEVW | 16 |
|  |  |  | 44 | 74 | HGRGGGDILEEEVWNRKSPIERLRECSRGCE | 31 |
|  |  |  | 76 | 100 | QHGEQREECLRRCQEEYQREKGRQD | 25 |
|  |  |  | 80 | 104 | QREECLRRCQEEYQREKGRQDDDNP | 25 |
|  |  |  | 92 | 115 | YQREKGRQDDDNPTDPEKQYQQCR | 24 |
|  |  |  | 96 | 120 | KGRQDDDNPTDPEKQYQQCRLQCRR | 25 |
|  |  |  | 106 | 127 | DPEKQYQQCRLQCRRQGEGGGF | 22 |
|  |  |  | 107 | 116 | PEKQYQQCRL | 10 |
|  |  |  | 108 | 142 | EKQYQQCRLQCRRQGEGGGFSREHCERRREEKYRE | 35 |
|  |  |  | 112 | 152 | QQCRLQCRRQGEGGGFSREHCERRREEKYREQQGREGGRGE | 41 |
|  |  |  | 114 | 129 | CRLQCRRQGEGGGFSR | 16 |
|  |  |  | 133 | 142 | ERRREEKYRE | 10 |
|  |  |  | 152 | 176 | EMYEGREREEEQEEQGRGRIPYVFE | 25 |
|  |  |  | 154 | 157 | YEGR | 4 |
|  |  |  | 164 | 171 | EEQGRGRI | 8 |
|  |  |  | 210 | 236 | YRVAILEAEPQTFIVPNHWDAESVVFV | 27 |
|  |  |  | 248 | 277 | QDRRESLNIKQGDILKINAGTTAYLINRDN | 30 |
|  |  |  | 258 | 281 | QGDILKINAGTTAYLINRDNNERL | 24 |
|  |  |  | 265 | 304 | NAGTTAYLINRDNNERLVLAKLLQPVSTPGEFELFFGAGG | 40 |
|  |  |  | 289 | 317 | PVSTPGEFELFFGAGGENPESFFKSFSDE | 29 |
|  |  |  | 309 | 323 | SFFKSFSDEILEAAF | 15 |
|  |  |  | 312 | 326 | KSFSDEILEAAFNTR | 15 |
|  |  |  | 351 | 367 | RAMSRHEEGGIWPFGGE | 17 |
|  |  |  | 352 | 367 | AMSRHEEGGIWPFGGE | 16 |
|  |  |  | 355 | 369 | RHEEGGIWPFGGESK | 15 |
|  |  |  | 362 | 366 | WPFGG | 5 |
|  |  |  | 362 | 366 | WPFGG | 5 |
|  |  |  | 377 | 410 | QRPTHSNQYGQLHEVDASQYRQLRDLDLTVSLAN | 34 |
|  |  |  | 411 | 448 | ITQGAMTAPHYNSKATKIALVVDGEGYFEMACPHMSRS | 38 |
|  |  |  | 429 | 447 | ALVVDGEGYFEMACPHMSR | 19 |
|  |  |  | 433 | 459 | DGEGYFEMACPHMSRSRGSYQGETRGR | 27 |
|  |  |  | 439 | 453 | EMACPHMSRSRGSYQ | 15 |
|  |  |  | 486 | 519 | VASSNQNLQVLCFEVNANNNEKFPLAGRRNVMNQ | 34 |
|  |  |  | 494 | 523 | QVLCFEVNANNNEKFPLAGRRNVMNQLERE | 30 |
|  |  |  | 497 | 529 | CFEVNANNNEKFPLAGRRNVMNQLEREAKELAF | 33 |
|  |  |  | 515 | 537 | NVMNQLEREAKELAFGMPAREVE | 23 |
|  |  |  | 530 | 541 | GMPAREVEEVSR | 12 |
|  |  |  | 533 | 549 | AREVEEVSRSQQEEFFF | 17 |
| 11S globulin seed storage protein 2 | Q9XHP0 | 28 | 8 | 11 | PSLR | 4 |
|  |  |  | 17 | 27 | GTTELWDERQE | 11 |
|  |  |  | 18 | 34 | TTELWDERQEQFQCAGI | 17 |
|  |  |  | 22 | 39 | WDERQEQFQCAGIVAMRS | 18 |
|  |  |  | 23 | 42 | DERQEQFQCAGIVAMRSTIR | 20 |
|  |  |  | 39 | 46 | STIRPNGL | 8 |
|  |  |  | 55 | 90 | PRLVYIERGQGLISIMVPGCAETYQVHRSQRTMERT | 36 |
|  |  |  | 60 | 76 | IERGQGLISIMVPGCAE | 17 |
|  |  |  | 67 | 82 | ISIMVPGCAETYQVHR | 16 |
|  |  |  | 71 | 84 | VPGCAETYQVHRSQ | 14 |
|  |  |  | 84 | 89 | QRTMER | 6 |
|  |  |  | 85 | 123 | RTMERTEASEQQDRGSVRDLHQKVHRLRQGDIVAIPSGA | 39 |
|  |  |  | 162 | 175 | GVPRSGEQEQQARQ | 14 |
|  |  |  | 169 | 186 | QEQQARQTFHNIFRAFDA | 18 |
|  |  |  | 218 | 234 | ERMTFVRPDEEEGEQEH | 17 |
|  |  |  | 219 | 235 | RMTFVRPDEEEGEQEHR | 17 |
|  |  |  | 226 | 260 | DEEEGEQEHRGRQLDNGLEETFCTMKFRTNVESRR | 35 |
|  |  |  | 236 | 256 | GRQLDNGLEETFCTMKFRTNV | 21 |
|  |  |  | 243 | 253 | LEETFCTMKFR | 11 |
|  |  |  | 251 | 273 | KFRTNVESRREADIFSRQAGRVH | 23 |
|  |  |  | 255 | 268 | NVESRREADIFSRQ | 14 |
|  |  |  | 261 | 270 | EADIFSRQAG | 10 |
|  |  |  | 290 | 322 | AEKGNLYSNALVSPDWSMTGHTIVYVTRGDAQV | 33 |
|  |  |  | 298 | 320 | NALVSPDWSMTGHTIVYVTRGDA | 23 |
|  |  |  | 333 | 359 | MNDRVNQGEMFVVPQYYTSTARAGNNG | 27 |
|  |  |  | 341 | 362 | EMFVVPQYYTSTARAGNNGFEW | 22 |
|  |  |  | 349 | 373 | YTSTARAGNNGFEWVAFKTTGSPMR | 25 |
|  |  |  | 360 | 373 | FEWVAFKTTGSPMR | 14 |
|  |  |  | 108 | 131 | DEYLIAGIDEINRTFELSPSWYIE | 24 |
|  |  |  | 115 | 148 | IDEINRTFELSPSWYIEALKYIKANHGLSGDAAT | 34 |
| Oleosin H1 | Q9FUJ9 | 7 | 2 | 39 | DRDRPHPHQIQVHPQHPHRYEGGVKSLLPQKGPSTTQI | 38 |
|  |  |  | 9 | 24 | HQIQVHPQHPHRYEGG | 16 |
|  |  |  | 39 | 70 | ILAIITLLPISGTLLCLAGITLVGTLIGLAVA | 32 |
|  |  |  | 87 | 103 | LIAGAVTAFLTSGAFGL | 17 |
|  |  |  | 105 | 125 | GLSSLSWVLNSFRRATGQGPL | 21 |
|  |  |  | 133 | 149 | QEGTLYVGEKTKQAGEA | 17 |
|  |  |  | 147 | 158 | GEAIKSTAKEGG | 12 |
| Oleosin L | Q9XHP2 | 3 | 17 | 20 | LQPR | 4 |
|  |  |  | 106 | 118 | PPGADQLESAKTK | 13 |
|  |  |  | 107 | 116 | PGADQLESAK | 10 |
| 2S albumin | Q9AUD1 | 18 | 18 | 40 | QQSQQCRQQLQGRQFRSCQRYLS | 23 |
|  |  |  | 21 | 44 | QQCRQQLQGRQFRSCQRYLSQGRS | 24 |
|  |  |  | 26 | 39 | QLQGRQFRSCQRYL | 14 |
|  |  |  | 29 | 53 | GRQFRSCQRYLSQGRSPYGGEEDEV | 25 |
|  |  |  | 38 | 71 | YLSQGRSPYGGEEDEVLEMSTGNQQSEQSLRDCC | 34 |
|  |  |  | 41 | 62 | QGRSPYGGEEDEVLEMSTGNQQ | 22 |
|  |  |  | 47 | 75 | GGEEDEVLEMSTGNQQSEQSLRDCCQQLR | 29 |
|  |  |  | 56 | 68 | MSTGNQQSEQSLR | 13 |
|  |  |  | 62 | 94 | QSEQSLRDCCQQLRNVDERCRCEAIRQAVRQQQ | 33 |
|  |  |  | 64 | 81 | EQSLRDCCQQLRNVDERC | 18 |
|  |  |  | 67 | 97 | LRDCCQQLRNVDERCRCEAIRQAVRQQQQEG | 31 |
|  |  |  | 68 | 85 | RDCCQQLRNVDERCRCEA | 18 |
|  |  |  | 69 | 75 | DCCQQLR | 7 |
|  |  |  | 70 | 84 | CCQQLRNVDERCRCE | 15 |
|  |  |  | 76 | 110 | NVDERCRCEAIRQAVRQQQQEGGYQEGQSQQVYQR | 35 |
|  |  |  | 81 | 97 | CRCEAIRQAVRQQQQEG | 17 |
|  |  |  | 95 | 120 | QEGGYQEGQSQQVYQRARDLPRRCNM | 26 |
|  |  |  | 117 | 129 | RCNMRPQQCQFRV | 13 |
| 2S albumin | Q9XHP1 | 15 | 10 | 44 | SVAEEGEEENQRGCEWESRQCQMRHCMQWMRSMRG | 35 |
|  |  |  | 13 | 33 | EEGEEENQRGCEWESRQCQMR | 21 |
|  |  |  | 17 | 34 | EENQRGCEWESRQCQMRH | 18 |
|  |  |  | 24 | 33 | EWESRQCQMR | 10 |
|  |  |  | 26 | 60 | ESRQCQMRHCMQWMRSMRGQYEESFLRSAEANQGQ | 35 |
|  |  |  | 31 | 39 | QMRHCMQWM | 9 |
|  |  |  | 43 | 78 | RGQYEESFLRSAEANQGQFEHFRECCNELRDVKSHC | 36 |
|  |  |  | 47 | 72 | EESFLRSAEANQGQFEHFRECCNELR | 26 |
|  |  |  | 56 | 74 | ANQGQFEHFRECCNELRDV | 19 |
|  |  |  | 60 | 92 | QFEHFRECCNELRDVKSHCRCEALRCMMRQMQQ | 33 |
|  |  |  | 65 | 89 | RECCNELRDVKSHCRCEALRCMMRQ | 25 |
|  |  |  | 68 | 93 | CNELRDVKSHCRCEALRCMMRQMQQE | 26 |
|  |  |  | 73 | 90 | DVKSHCRCEALRCMMRQM | 18 |
|  |  |  | 75 | 104 | KSHCRCEALRCMMRQMQQEYGMEQEMQQMQ | 30 |
|  |  |  | 119 | 134 | MQQMQQMMQYLPRMCG | 16 |

* Sequence start and end are referred to sesame (*Sesamum indicum*) proteins (associated with the Uniprot accession number given) without considering the pro-peptide.

**Table S3**. Experimentally validated linear IgE-binding epitopes from *Sesamum indicum* allergens used for the peptide conservancy analysis. Table A: Ses i 2 (Q9XHP1) and Table B Ses i 5 (Q9XHP2)

**A.**

| **N°** | **Epitope** | **IEDB ID^a^** | **Starting Position** | **Ending Position** | **Source**  **Molecule** | **Allergen** | **Uniprot**  **code** |
| --- | --- | --- | --- | --- | --- | --- | --- |
| 1 | CMQWMRSMRG | 6663 | 54 | 63 | 2S albumin | Ses i 2 | Q9XHP1 |
| 2 | EANQGQFEHF | 11149 | 74 | 83 | 2S albumin | Ses i 2 | Q9XHP1 |
| 3 | FEHFRECCNE | 15547 | 80 | 89 | 2S albumin | Ses i 2 | Q9XHP1 |
| 4 | GEEENQRGCEWESRQCQMRH | 19237 | 34 | 53 | 2S albumin | Ses i 2 | Q9XHP1 |
| 5 | GQFEHFRECC | 21860 | 78 | 87 | 2S albumin | Ses i 2 | Q9XHP1 |
| 6 | NQGQFEHFREC | 45561 | 76 | 86 | 2S albumin | Ses i 2 | Q9XHP1 |
| 7 | QCQMRHCM | 50419 | 48 | 55 | 2S albumin | Ses i 2 | Q9XHP1 |
| 8 | QYEESFLRSAEANQGQFEHFREC | 52895 | 64 | 86 | 2S albumin | Ses i 2 | Q9XHP1 |
| 9 | TVVTTSVAEEGEEENQRGCE | 67208 | 24 | 43 | 2S albumin | Ses i 2 | Q9XHP1 |

**B.**

| **N°** | **Epitope** | **IEDB ID^a^** | **Starting Position** | **Ending Position** | **Source**  **Molecule** | **Allergen** | **Uniprot**  **code** |
| --- | --- | --- | --- | --- | --- | --- | --- |
| 1 | APHLQLQPRAQR | 1863025 | 13 | 24 | Oleosin L | Ses i 5 | Q9XHP2 |
| 2 | AEQFSQQPVAGSQTS | 2271912 | 131 | 145 | Oleosin L | Ses i 5 | Q9XHP2 |
| 3 | ALSVLSWIYRYLTGK | 2271921 | 91 | 105 | Oleosin L | Ses i 5 | Q9XHP2 |
| 4 | ASKAREMKDRAEQFS | 2271926 | 121 | 135 | Oleosin L | Ses i 5 | Q9XHP2 |
| 5 | AVTAGGSLLVLSGLT | 2271930 | 31 | 45 | Oleosin L | Ses i 5 | Q9XHP2 |
| 6 | DQLESAKTKLASKAR | 2271937 | 111 | 125 | Oleosin L | Ses i 5 | Q9XHP2 |
| 7 | FLASGGFGVAALSVL | 2271954 | 81 | 95 | Oleosin L | Ses i 5 | Q9XHP2 |
| 8 | MAEHYGQQQQTRAPH | 2272069 | 1 | 15 | Oleosin L | Ses i 5 | Q9XHP2 |
| 9 | RAQRVVKAATAVTAG | 2272130 | 21 | 35 | Oleosin L | Ses i 5 | Q9XHP2 |
| 10 | TRAPHLQLQPRAQRV | 2272183 | 11 | 25 | Oleosin L | Ses i 5 | Q9XHP2 |
| 11 | VIFSPVLVPAVITIF | 2272188 | 61 | 75 | Oleosin L | Ses i 5 | Q9XHP2 |
| 12 | VITIFLLGAGFLASG | 2272189 | 71 | 85 | Oleosin L | Ses i 5 | Q9XHP2 |
| 13 | YLTGKHPPGADQLES | 2272232 | 101 | 115 | Oleosin L | Ses i 5 | Q9XHP2 |

^a^Epitope information was retrieved from the Immune Epitope Database (IEDB, https://www.iedb.org/) searching for *Sesamum indicum* organism.

**2. Supplementary Figures**


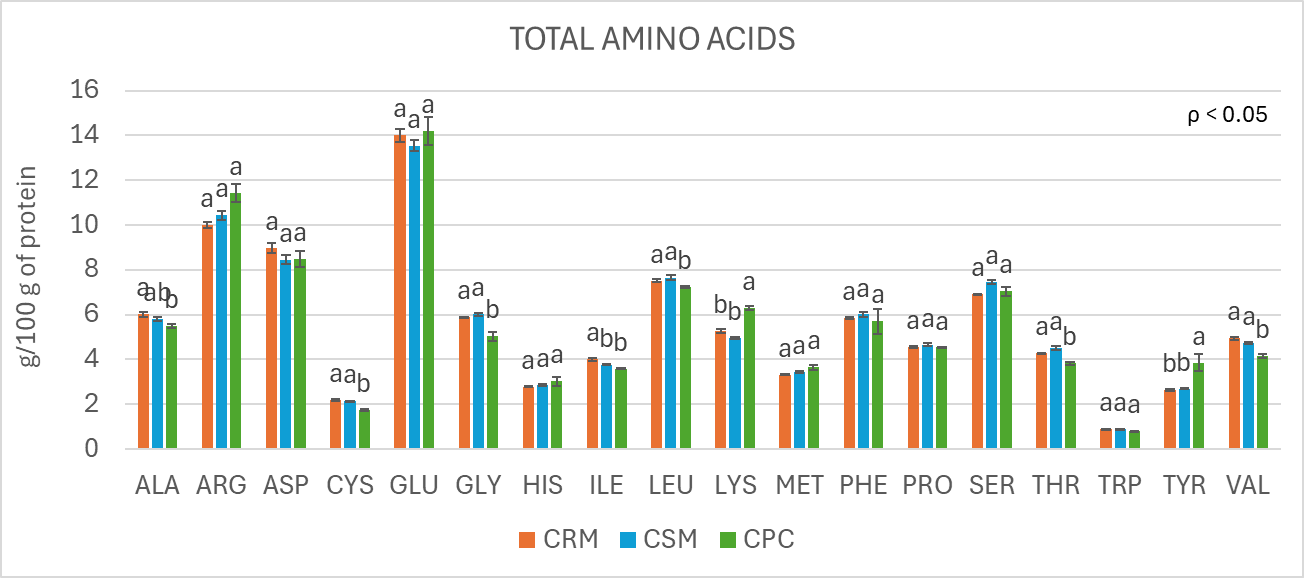


**Figure S1**. Amino acid profile of chia samples – chia raw material (CRM) in orange, chia standardized raw material (CSM) in light blue, and chia protein concentrate (CPC) in green – expressed as g/100 g of protein. Letters refers to statistical differences between samples (one-way ANOVA; Tukey’s test, ρ < 0.05).


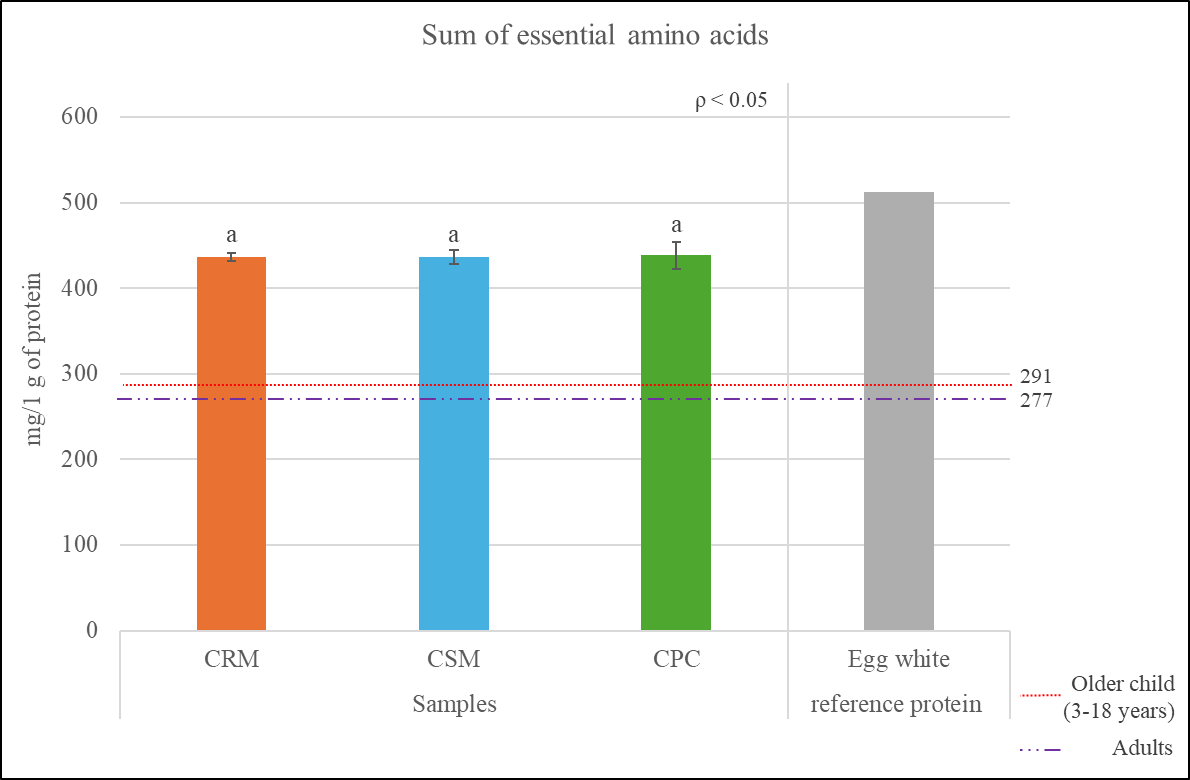


**Figure S2**. Sum of essential amino acids of chia samples – chia raw material (CRM) in orange, chia standardized raw material (CSM) in light blue, and chia protein concentrate (CPC) in green – compared with the egg white reference protein (in grey), expressed as g/100 g of protein. Letters refers to statistical differences between samples (one-way ANOVA; Tukey’s test, ρ < 0.05).


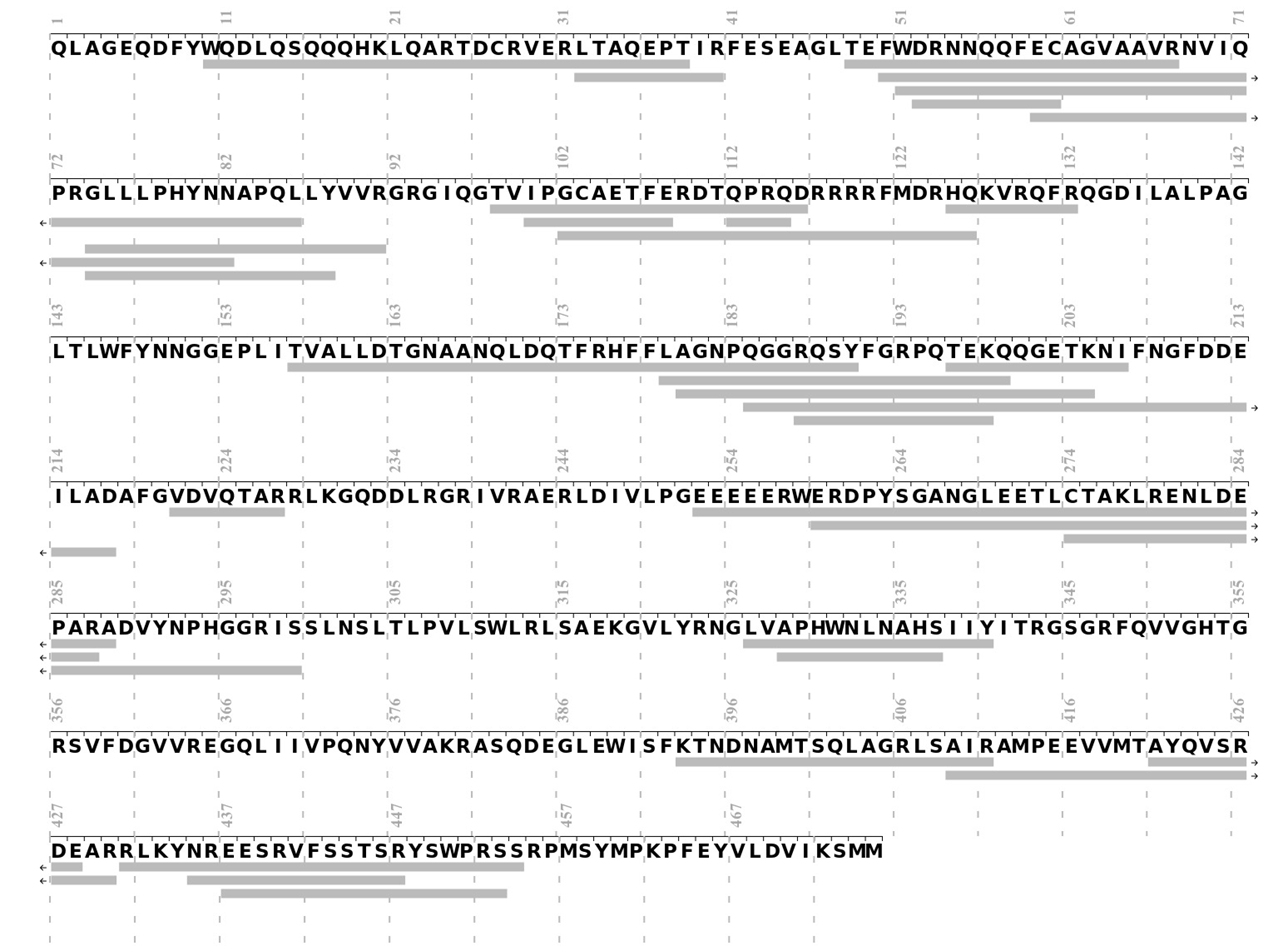


**Figure S3**. Graphically elucidated coverage of 11S globulin precursor (Q9AUD2)


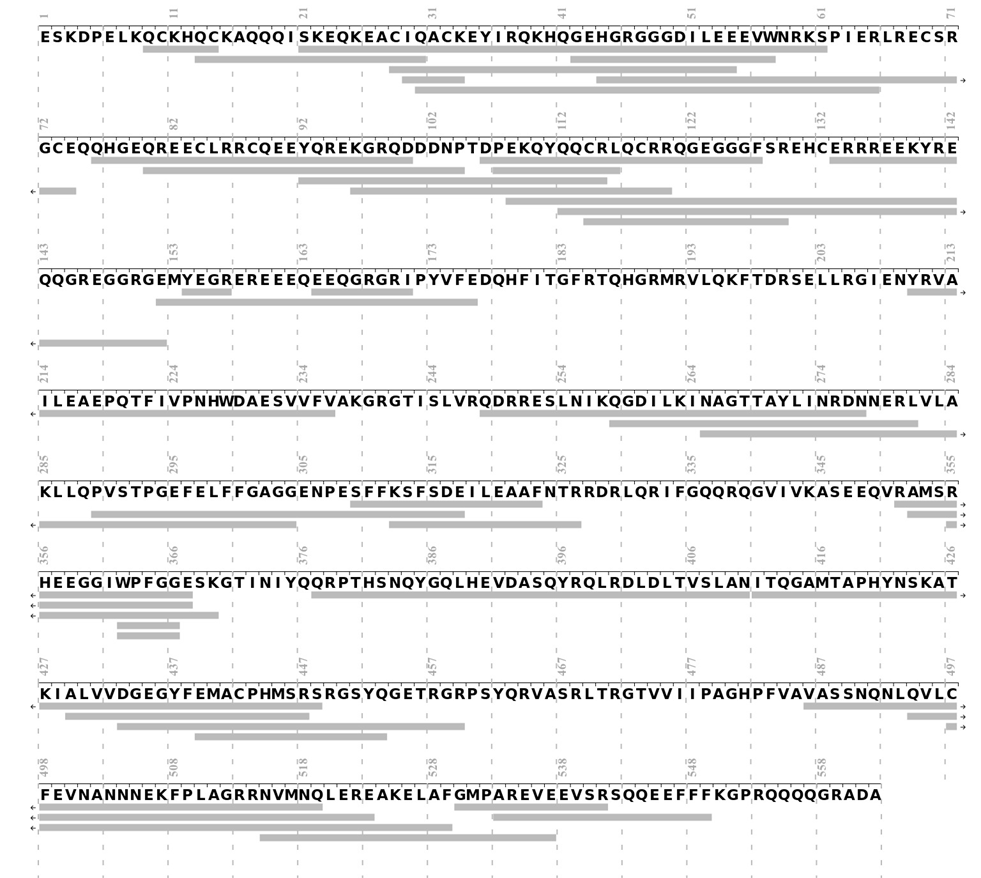


**Figure S4**. Graphically elucidated coverage of 7S globulin (Q9AUD0)


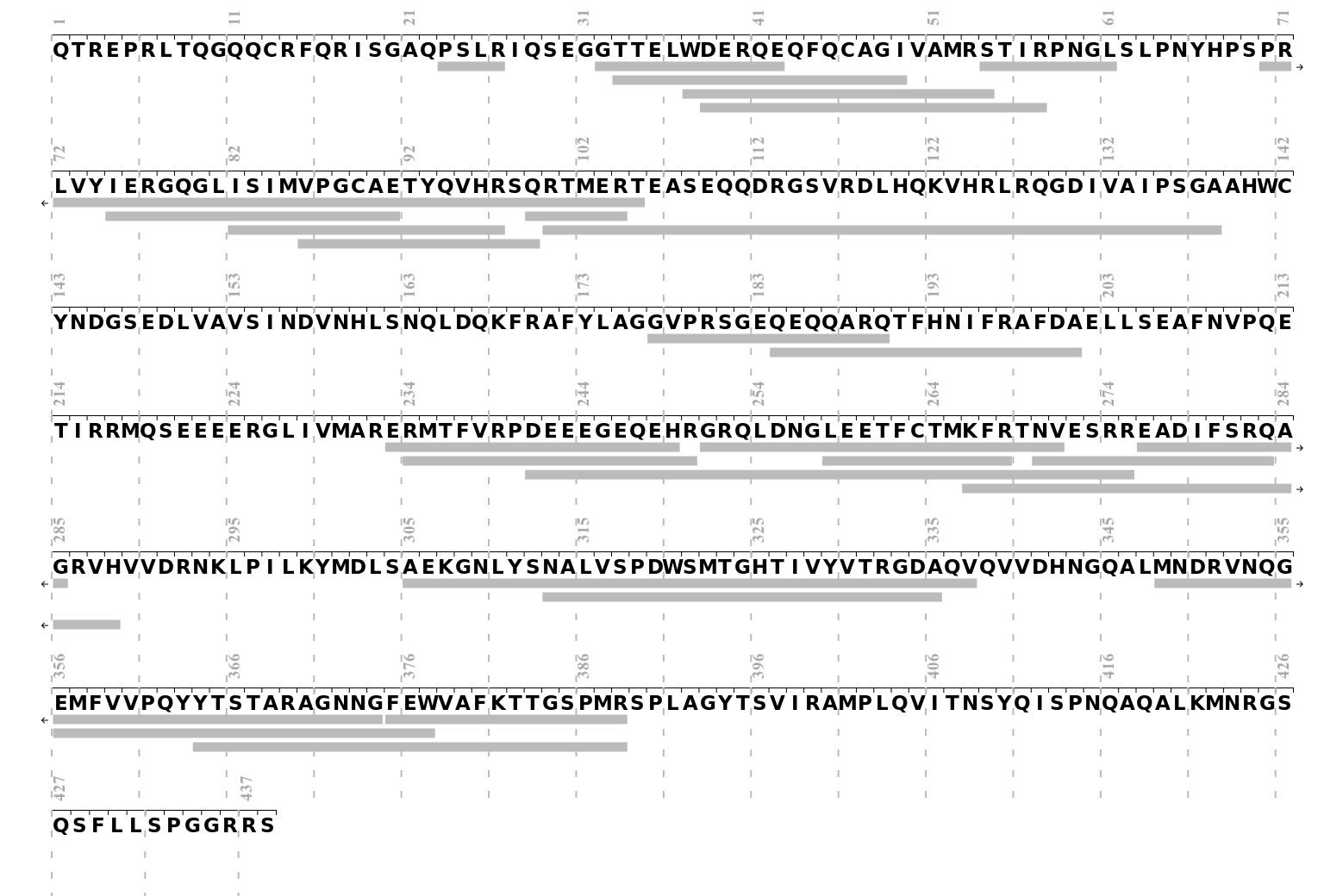


**Figure S5**. Graphically elucidated coverage of 11S globulin seed storage protein 2 (Q9XHP0)


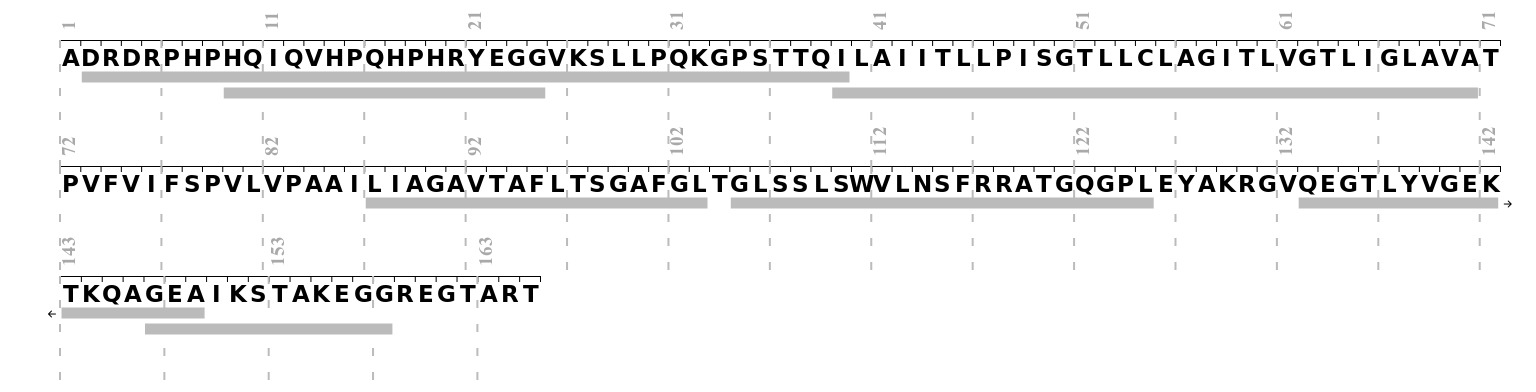


**Figure S6**. Graphically elucidated coverage of Oleosin H1 (Q9FUJ9)


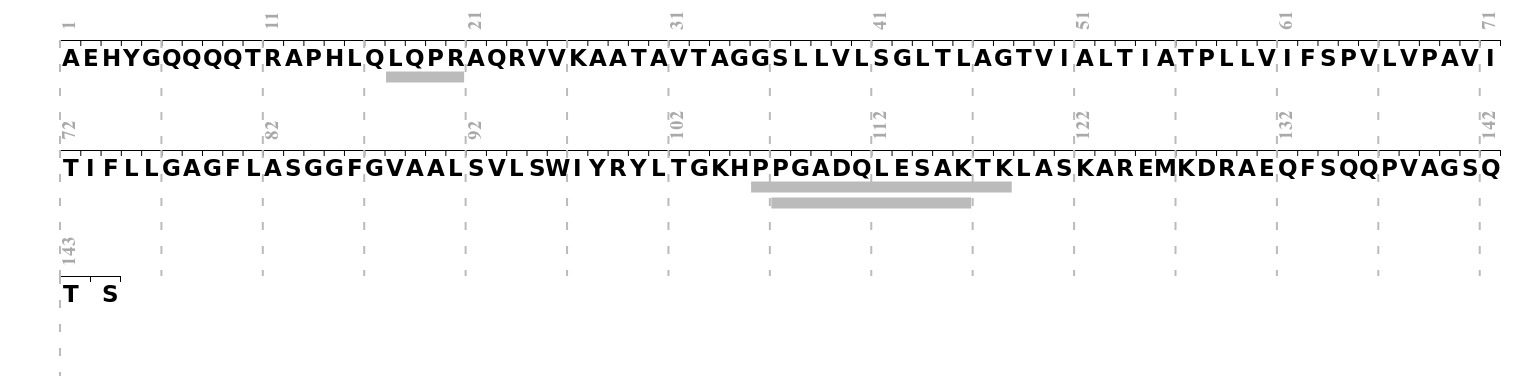


**Figure S7**. Graphically elucidated coverage of Oleosin L (Q9XHP2)


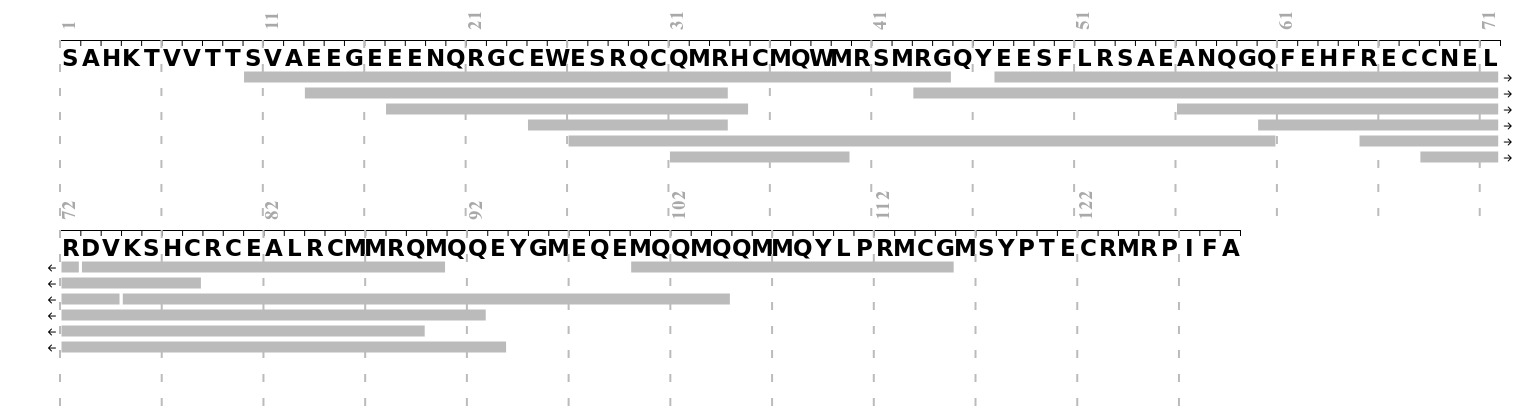


**Figure S8**. Graphically elucidated coverage of 2S seed storage protein 1 (Q9XHP1)


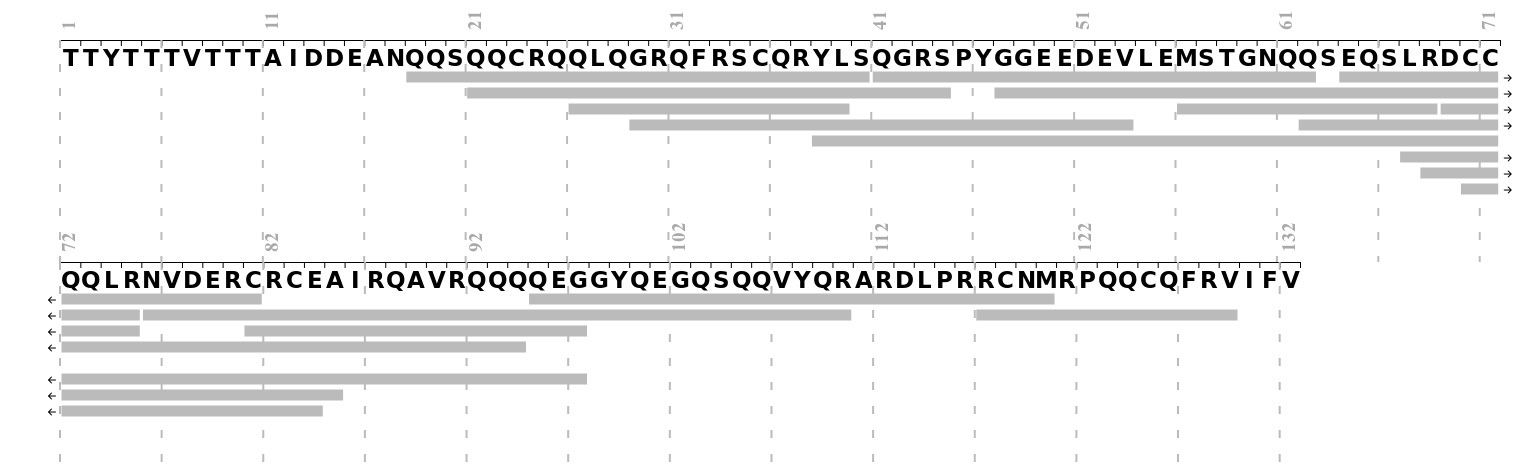


**Figure S9**. Graphically elucidated coverage of 2S albumin (Q9AUD1)

References

1. Santos AF, Riggioni C, Agache I, et al. EAACI guidelines on the diagnosis of IgE-mediated food allergy. Allergy. 2023; 78: 3057-3076. doi:10.1111/all.15902
